# 16S-ITGDB: An Integrated Database for Improving Species Classification of Prokaryotic 16S Ribosomal RNA Sequences

**DOI:** 10.3389/fbinf.2022.905489

**Published:** 2022-08-03

**Authors:** Yu-Peng Hsieh, Yuan-Mao Hung, Mong-Hsun Tsai, Liang-Chuan Lai, Eric Y. Chuang

**Affiliations:** ^1^ Department of Electrical Engineering, National Taiwan University, Taipei, Taiwan; ^2^ Graduate Institute of Biomedical Electronics and Bioinformatics, National Taiwan University, Taipei, Taiwan; ^3^ Institute of Biotechnology, National Taiwan University, Taipei, Taiwan; ^4^ Bioinformatics and Biostatistics Core, Center of Genomic and Precision Medicine, National Taiwan University, Taipei, Taiwan; ^5^ Graduate Institute of Physiology, College of Medicine, National Taiwan University, Taipei, Taiwan; ^6^ College of Biomedical Engineering, China Medical University, Taichung, Taiwan

**Keywords:** taxonomy assignment, 16S full length, ITGDB, sequence classification, 16S rRNA (16S rDNA), metagenomics 16S, third-generation sequencing

## Abstract

Analyzing 16S ribosomal RNA (rRNA) sequences allows researchers to elucidate the prokaryotic composition of an environment. In recent years, third-generation sequencing technology has provided opportunities for researchers to perform full-length sequence analysis of bacterial 16S rRNA. RDP, SILVA, and Greengenes are the most widely used 16S rRNA databases. Many 16S rRNA classifiers have used these databases as a reference for taxonomic assignment tasks. However, some of the prokaryotic taxonomies only exist in one of the three databases. Furthermore, Greengenes and SILVA include a considerable number of taxonomies that do not have the resolution to the species level, which has limited the classifiers’ performance. In order to improve the accuracy of taxonomic assignment at the species level for full-length 16S rRNA sequences, we manually curated the three databases and removed the sequences that did not have a species name. We then established a taxonomy-based integrated database by considering both taxonomies and sequences from all three 16S rRNA databases and validated it by a mock community. Results showed that our taxonomy-based integrated database had improved taxonomic resolution to the species level. The integrated database and the related datasets are available at https://github.com/yphsieh/ItgDB.

## 1 Introduction

Since the advent of next-generation sequencing (NGS) technology, analyzing 16S ribosomal RNA (rRNA) has allowed biologists to assess the bacterial or archaeal composition of an environment. The 16S rRNA gene consists of nine hypervariable regions (V1–V9) and includes approximately 1,500 ∼1,600 nucleotides (Bukin et al., 2019; Johnson et al., 2019). These regions have varying conservation and are rich in taxonomic information. Different hypervariable regions were investigated to improve the taxonomic assignment performance (Wang and Qian, 2009; Allard et al., 2015; Yang et al., 2016; Bukin et al., 2019; Johnson et al., 2019; Abellan-Schneyder et al., 2021). In the past decade, the 16S rRNA V4 or V3–V4 regions were targeted for microbial composition analysis (Richards et al., 2017; Jha et al., 2018; Moustafa et al., 2018; Peters et al., 2018). However, NGS technology generated short reads that covered only a few 16S rRNA regions (Yang et al., 2016). Using only one or two hypervariable regions makes it difficult to classify the bacterial 16S rRNA sequences down to the species level in taxonomic assignment tasks (Johnson et al., 2019). For a prokaryotic 16S sequence classifier, it requires at least 400 nucleotides to assign a 16S sequence down to the genus level (Okubo et al., 2009). However, after quality control, the read length of the trimmed 16S sequences was about 250 ∼500 base-pairs (bp), which limits the taxonomic resolution only to the genus levels. Thus, full-length 16S rRNA sequence analysis could be the resolution to improve the taxonomic depth down to the species level.

In recent years, third-generation sequencing (TGS) technology, such as Pacific BioScience (PacBio) (Rhoads and Au, 2015; Schloss et al., 2016) and Nanopore (Lu et al., 2016; Lin et al., 2021), has provided long-read sequencing methods, making it possible for researchers to analyze the full-length of 16S rRNA (Cuscó et al., 2018; Klemetsen et al., 2019). The full-length sequence analysis could enhance taxonomic resolution to the species level because the long reads that include the V1–V9 regions provide more comprehensive taxonomic information (Johnson et al., 2019). The single-molecule real-time (SMRT) and circular consensus sequencing (CCS) technologies developed by PacBio could provide high quality 16S full-length sequencing (Korlach, 2013). During the past 5 years, a growing number of studies took the advantage of long read sequencing technology to attain more comprehensive microbial composition of the environments (Hur and Park, 2019; Tremblay and Yergeau, 2019; Lam et al., 2020; Wade and Prosdocimi, 2020; Mahmud et al., 2021; Pootakham et al., 2021). However, although there were several widely used 16S analytical pipelines for NGS data analysis, such as QIIME2 (Bolyen et al., 2019), Mothur (Schloss, 2020), and UPARSE (Edgar, 2013), there still lacks comprehensive and convenient 16S tools for TGS data analysis. Researchers may need to build their own 16S full-length analytical pipeline. Yet, the advantages of 16S full-length sequence analysis could only be demonstrated when the taxonomic assignment tools, including 16S rRNA classifiers and sequence databases, are well prepared.

Several classification algorithms have been proposed to classify bacterial 16S rRNA sequences (Wang et al., 2007; Allard et al., 2015; Edgar, 2016; Bokulich et al., 2018; Schloss, 2020). These classification algorithms used prokaryotic 16S databases, such as the ribosomal database project (RDP) (Maidak et al., 1997), SILVA (Quast et al., 2012), or Greengenes (DeSantis et al., 2006), as references. The RDP and SILVA databases are still being updated regularly, whereas Greengenes was not updated after August of 2013. Therefore, Greengenes includes fewer bacterial species than RDP and SILVA.

Next, regarding these 16S rRNA databases, some taxonomies have annotated to the species level, while others may only include information to the genus, family, order, class, or even just phylum level. Even among the sequences with taxonomic information at the species level, the species information does not always have exact species name (sometimes the species names are listed as metagenome, candidate_division, bacterium, etc.). Sequences with anomalous nucleotide composition or labeled with low-resolution taxonomy dramatically limits the performance of classifiers. Furthermore, RDP, SILVA, and Greengenes have their own unique taxonomies (Abellan-Schneyder et al., 2021; Balvočiūtė and Huson, 2017), and it is impossible for a classifier to identify the bacterial taxonomy from these three databases other than the reference database used to establish the classifier. Therefore, in order to improve the classification performance, the 16S rRNA integrated database (ITGDB) was developed in this study by two ways: sequence-based and taxonomy-based integration. Both of the integrated databases were compared with RDP, SILVA, Greengenes, and other curated 16S reference databases, including 16S-UDb (Agnihotry et al., 2020), Genomic-based 16S rRNA database (Abellan-Schneyder et al., 2021), and Genome taxonomy database (Parks et al., 2021). The integrated database (ITGDB) can be used for any classifier that was developed in a specific reference database and largely improved the assignment resolution to the species level. The proposed 16S rRNA integrated databases can be downloaded from https://github.com/yphsieh/ItgDB.

## 2 Materials and Methods

RDP (version NO.18 trainset) (Maidak et al., 1997), SILVA (version 138, 99% clustering similarity) (Quast et al., 2012), and Greengenes (version 13_8, 99% clustering similarity) (DeSantis et al., 2006) databases were used for integration. Redundant sequences were removed by clustering all the sequences in these databases with 99% similarity. The sequence numbers of RDP, SILVA, and Greengenes were 21,295, 436,681, and 203,452, respectively. The percentages of the sequences that had exact species names in RDP, SILVA, and Greengenes were 94.86, 16.10, and 10.19%, respectively. Among these databases, RDP had the smallest quantity of sequences but possessed the highest percentage of sequences with exact species names. SILVA had the largest quantity of sequences, but most of the sequences did not have taxonomic resolution to the species level. The sequences without exact species names were manually removed from the databases.

**FIGURE 1:**
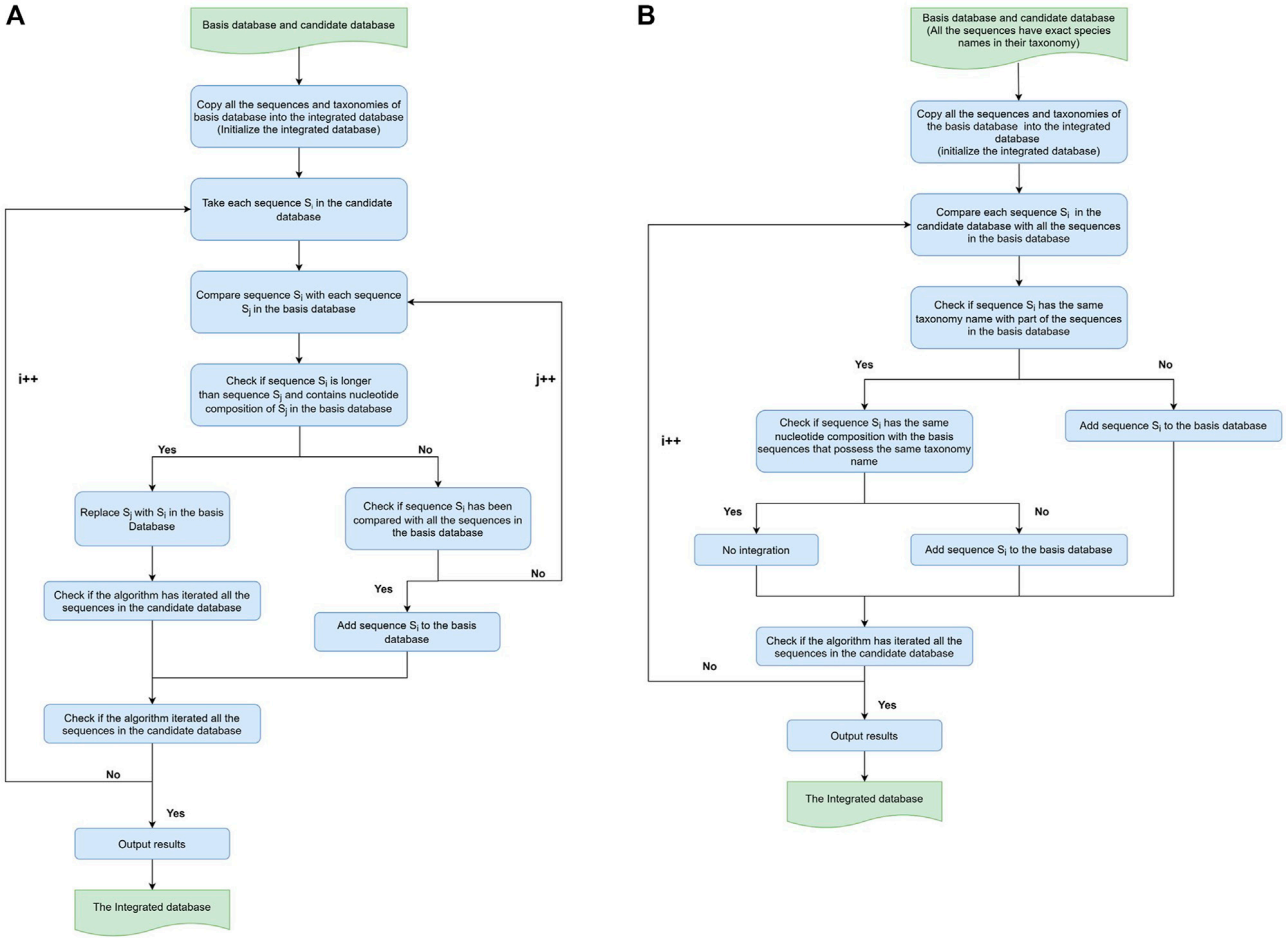
Workflow of sequence-based and taxonomy-based Integration. **(A)** Sequence-based integration and **(B)** taxonomy-based integration. For both sequence-based integration and taxonomy-based integration workflows, the algorithm took RDP as the basis database and SILVA as the candidate database in the first round to produce the intermediate RS-ITGDB. Then, the algorithm took RS-ITGDB (new basis) and Greengenes (new candidate) as inputs to run this workflow again and produce the final integrated database.

In our integration workflow, since RDP and SILVA included the newest information on bacteria and archaea, these two databases were firstly integrated. This integration produced an intermediate database—RDP and SILVA integrated database (RS-ITGDB). Next, the intermediate RS-ITGDB was further integrated with the Greengenes database. There were two types of integration—sequence-based integration and taxonomy-based integration (Figure 1). Both integrations were developed by using Python scripts. The algorithms were described as follows.

### 2.1 Sequence-Based Integration

The concept of sequence-based integration was to collect all the sequences from RDP, SILVA, and Greengenes, regardless of the quality of taxonomic annotation. The workflow of sequence-based integration of any two databases (called the ‘basis’ database and the ‘candidate’ database) is shown in Figure 1 **(A)**. The algorithm first took RDP as the basis database and integrated RDP with SILVA to produce the intermediate RDP-SILVA integrated database (RS-ITGDB). Next, the algorithm took RS-ITGDB as the basis database and integrated RS-ITGDB with Greengenes to produce the final sequence-based integrated database (ITGDB). During the sequence-based integration, the algorithm checked whether each sequence *S*_*i*_ in the candidate database already existed in the basis database by comparing the nucleotide composition between the sequences. If the nucleotide composition of sequence *S*_*i*_ contained the nucleotide composition of a sequence *S*_*j*_ from the basis database, i.e., *S*_*i*_ was longer than *S*_*j*_, then sequence *S*_*j*_ would be replaced with sequence *S*_*i*_ in the integrated database. If sequence *S*_*i*_ could not be found in the basis database, then sequence *S*_*i*_ would be directly added to the integrated database. Sequences *S*_*i*_ and *S*_*j*_ were regarded as different sequences (not contain each other) even if they only had one nucleotide difference. The algorithm terminated after comparing all the sequences between the basis database and candidate database.

### 2.2 Taxonomy-Based Integration

For taxonomy-based integration, all sequences without exact species names were manually removed from RDP, SILVA, and Greengenes. For example, Acidocella_sp. only indicates the genus name with the abbreviation “sp.” in the species name. Some taxonomies only showed ambiguous description at the species level, such as “bacterium,” “metagenome,” “candidate_division,” “human_gut,” and “unidentified.” All sequences with such ambiguous species names were manually removed from the 16S databases to ensure each sequence had taxonomic resolution to the species level.

The concept of taxonomy-based integration was first to collect the unique taxonomy from RDP, SILVA, and Greengenes and then integrate the different sequences for each taxonomy. The workflow of taxonomy-based integration of any two databases is shown in Figure 1B. It is similar to the sequence-based integration. The algorithm first took RDP as the basis database and integrated RDP with SILVA to produce the intermediate RDP-SILVA integrated database (RS-ITGDB). Next, the algorithm took RS-ITGDB as the basis database and integrated RS-ITGDB with Greengenes to produce the final taxonomy-based integrating database. During the taxonomy-based integration procedure, if a sequence *S*_*i*_ from the candidate database had taxonomy that could not be found in the basis database, then sequence *S*_*i*_ was added to the integrated database. The algorithm checked whether the taxonomy of sequence *S*_*i*_ already existed in the basis database by comparing the string of taxonomic label of sequence *S*_*i*_ with all taxonomies in the basis database. If the taxonomy of sequence *S*_*i*_ already existed in the basis database, then the algorithm further compared the nucleotide composition between sequence *S*_*i*_ and all the sequences of the basis database that possess the same taxonomy as *S*_*i*_. If the nucleotide composition of *S*_*i*_ had at least one nucleotide difference with the sequences of the basis database under the same taxonomy, then sequence *S*_*i*_ was added to the integrated database. Inversely, if sequence *S*_*i*_ had already been collected in the basis database, no integration occurred.

### 2.3 Validation

Two experiments were carried out to validate the performance of the developed ITGDBs. One was database comparison, and the other was the ITGDBs’ performance with different classifiers. The purpose of the database comparison analysis was to compare the performance of our developed ITGDBs with other 16S reference databases. Another experiment was to measure the performance of several widely used 16S sequence classifiers using the ITGDB as the reference database.

#### 2.3.1 The Applied 16S Reference Databases

Our proposed sequence-based ITGDB and taxonomy-based ITGDB were compared with RPD, SILVA, Greengenes, and other manually curated 16S sequence datasets, such as 16S-UDb (Agnihotry et al., 2020), Genomic-based 16S rRNA database (GRD) (Abellan-Schneyder et al., 2021) (https://metasystems.riken.jp/grd/), and Genome taxonomy database (GTDB) (Parks et al., 2021). Part of the 16S-UDb content was curated from early versions of SILVA (version 123), Greengenes (version 13_5), and RDP (version 11.4) based on 97% similarity in OTU clustering threshold. The 16S sequences in the GRD dataset were curated from the complete genome sequences and had sequence length from 65 to 2,900 nucleotides (Desai et al., 2020). Each sequence in 16S-UDb and GRD had taxonomic information down to the species level. The sequence numbers of 16S-UDb and GRD were 13,078 and 13,202, respectively. GTDB is a comprehensive metagenomic database that curated prokaryotic genome and taxonomies from the NCBI Assembly database (Parks et al., 2021). GTDB also supported 16S rRNA sequences that were extracted from the genomic database (Alishum, 2021). The sequence number of GTDB 16S dataset was 32,884.

#### 2.3.2 Validation Datasets

The validation dataset for sequence-by-sequence validation was created by integrating the public mock communities, including Mockrobiota (Bokulich et al., 2016), PacBio HMP (Callahan et al., 2019), and PacBio Zymo (Callahan et al., 2019). First, unique sequences in 15 mock communities with comprehensive taxonomy information in Mockrobiota (Bokulich et al., 2016), such as mock 3, 4, 5, and 12 to 23, were used for the experiments. Next, PacBio HMP (Callahan et al., 2019) and PacBio Zymo (Callahan et al., 2019) mock communities were used, too. Since sequences in the PacBio HMP and Zymo mock community lacked taxonomy information, BLAST accompanied with the NCBI microbial 16S rRNA database was performed to annotate all sequences with species information (Bokulich et al., 2016). Finally, the validation dataset was created by combining Mockrobiota with the PacBio HMP and Zymo dataset. In total, the combined mock validation dataset contained 98,284 reads with taxonomy names to the species level in 94 species. The average sequence length was 1,548 bp.

**FIGURE 2:**
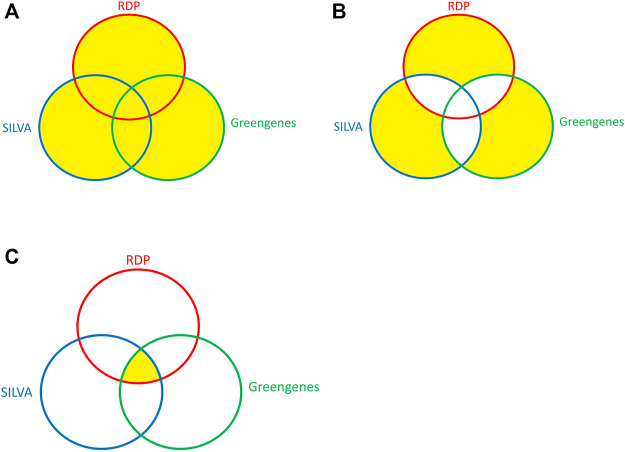
The Venn diagram of the Union, Exclusion, and Intersection datasets. **(A)** Union, **(B)** Exclusion, and **(C)** Intersection.

To test whether ITGDB had better performance in identifying the unique taxonomies than other three databases, another three validation datasets were prepared—Union, Exclusion, and Intersection. Among these datasets, Union and Exclusion were designed to collect the unique taxonomies from different databases, while the Intersection dataset was used to validate the performance of different reference databases without unique taxonomies. The concepts of producing Union, Exclusion, and Intersection datasets are shown in Figure 2.

All the sequences in the validation datasets had exact species names. The Union dataset contained all the available sequences with exact species names in any of the three source databases. The Exclusion dataset contained the sequences whose species names were only available in one of the databases. The Intersection dataset contained the sequences whose species names were present in all three databases.

#### 2.3.3 Classifiers

To assess the ITGDBs’ performance with compatible classifier experiments, we chose several widely used 16S classifiers: QIIME2 (RDP Bayesian classifier, version 2020.8) (Bokulich et al., 2018), SINTAX (usesarch version 11.0.667) (Edgar, 2016), SPINGO (version 1.3) (Allard et al., 2015), and Mothur (RDP Bayesian classifier, version 1.45.2) (Schloss, 2020).

For the database comparison analysis, SINTAX was used as the standard for taxonomic assignment because SINTAX provided more comprehensive assignment results. Just like other 16S RDP-like classifiers, SINTAX also calculated a confidence score for each taxonomic level and used confidence thresholds to filter out the taxonomic levels that had scores lower than the threshold. SINTAX provided both “cut-off” and “no cut-off” results for its users. The setting of /the SINTAX classifier for the “cut-off” results was 0.8 (default setting). The “no cut-off” results included the assignment information from the kingdom to the species level, and these results were used for validation to ensure that each sequence included species information. Given the 16S full-length reads provided by the third-generation sequencing technology include approximately 1,200 ∼1,500 nucleotides, the “no cut-off” assignment was applied in this study to assign the sequences to the species level.

#### 2.3.4 Validation Metrics

The validation metrics included accuracy, precision, recall, and F1-score, as shown in the following equations:

$$Accuracy=\frac{TP+TN}{TP+FP+TN+FN}$$ (1)

$$Precision=\frac{TP}{TP+FP}$$ (2)

$$Recall=\frac{TP}{TP+FN}$$ (3)

$$F1-score=\frac{2\times precision\times recall}{precision+recall}$$ (4)

where TP is true positive, FP is false positive, TN is true negative, and FN is false negative.

We measured all four metrics for each taxonomic level. For a classified sequence, if the assigned taxonomic name in a taxonomic level matched the name in the validation dataset’s corresponding level, it was regarded as a correct assignment for the taxonomic level. However, the scientific names in some databases were used to describe the microbial taxonomy, while others might apply different naming conventions (Federhen, 2012). This situation formed an obstacle to comparing the taxonomic names from phylum to the species levels. Therefore, NCBI taxonomy dump files (https://ftp.ncbi.nlm.nih.gov/pub/taxonomy/), which included scientific names and all possible synonyms of each taxonomic level for one microbial species, were applied to address this issue.

#### 2.3.5 Performance Comparison Between Reference Databases

SINTAX was used for taxonomy assignment in the database comparison experiment because SINTAX showed good performance in sequence classification and provided comprehensive assignment results (Hung et al., 2022). Each reference database, including RDP, SILVA, and Greengenes, was used as the SINTAX’s reference for taxonomic assignment tasks. The assignment results were compared with the correct taxonomies in the validation data to calculate the accuracy, precision, recall, and F1-score for comparison. Then, the performance of using different reference databases for taxonomic assignment was compared.

As mentioned before, SINTAX provided both “cut-off” and “no cut-off” assignment results. “No cut-off” taxonomies were applied to ensure the assignment results including species information. For the “cut-off” results, the cut-off value was set at 0.8 (default setting).

#### 2.3.6 Work With Different Classifiers

The performance of the widely used 16S sequence classifiers, such as SINTAX, SPINGO, Mothur, and QIIME2, was compared with our proposed integrated database. All the classifiers were set at default values and in “no cut-off” mode to ensure the assignment results to the species names. The settings of the SINTAX classifier were the same as described previously in Section 2.3.5. For the SPINGO classifier, the k-mer size and bootstrap value were set as 8 and 10 (default values). The Mothur classifier was set as “wang,” which was an RDP-like classification method. The k-mer size was 8 (default), and the cut-off value was set as 0. For the QIIME2 Bayesian classifier, the k-mer size parameter was set as 7 (default) and the confidence threshold value was set as “disable.” Accuracy, precision, recall, and F1-score were measured for each classifier.

## 3 Results

To enhance taxonomic assignment resolution, we manually curated RDP, SILVA, and Greengenes datasets and removed the sequences that did not have exact species names. In total, the numbers of sequences that were manually removed were 1,095 from RDP, 366,392 from SILVA, and 182,728 from Greengenes, respectively. The final numbers of sequences in the sequence-based and taxonomy-based ITGDBs were 486,640 and 110,780, respectively. For ITGDBs and the source databases, the sequence counts of the hypervariable regions for 16S metabarcoding studies are listed in Table 1. RDP and sequence-based ITGDB have the minimum (4,644) and maximum (113,460) V1-V9 sequences, respectively. Taxonomy-based ITGDB (34,639) has fewer number of V1-V9 sequences than SILVA (101,649), Greengenes (49,286), and sequence-based ITGDB (113,460) due to the removal of the sequences with blurred species information.

**TABLE 1:** The sequence number of the hypervariable regions in the source databases and ITGDB.

Regions	RDP	SILVA	Greengenes	Seq_ITGDB^a^	Taxa_ITGDB^b^
V1-V2	7,034	168,480	97,881	192,781	46,607
V1-V3	5,761	143,412	83,900	163,782	40,634
V3	20,551	411,072	197,086	459,207	107,675
V4	20,970	386,890	202,617	436,366	110,039
V3-V4	20,365	367,701	197,762	415,735	107,635
V3-V5	20,327	366,873	197,277	414,767	107,510
V4-V5	20,900	384,157	202,370	433,543	109,905
V6-V8	19,888	316,720	176,913	358,965	101,316
V6-V9	9,931	143,014	70,820	161,039	52,613
V7-V9	10,282	145,409	72,801	163,863	54,159
V1-V9	4,644	101,694	49,286	113,460	34,639

a Seq_ITGDB: sequence-based integrated database.

b Taxa_ITGDB: taxonomy-based integrated database.

**FIGURE 3:**
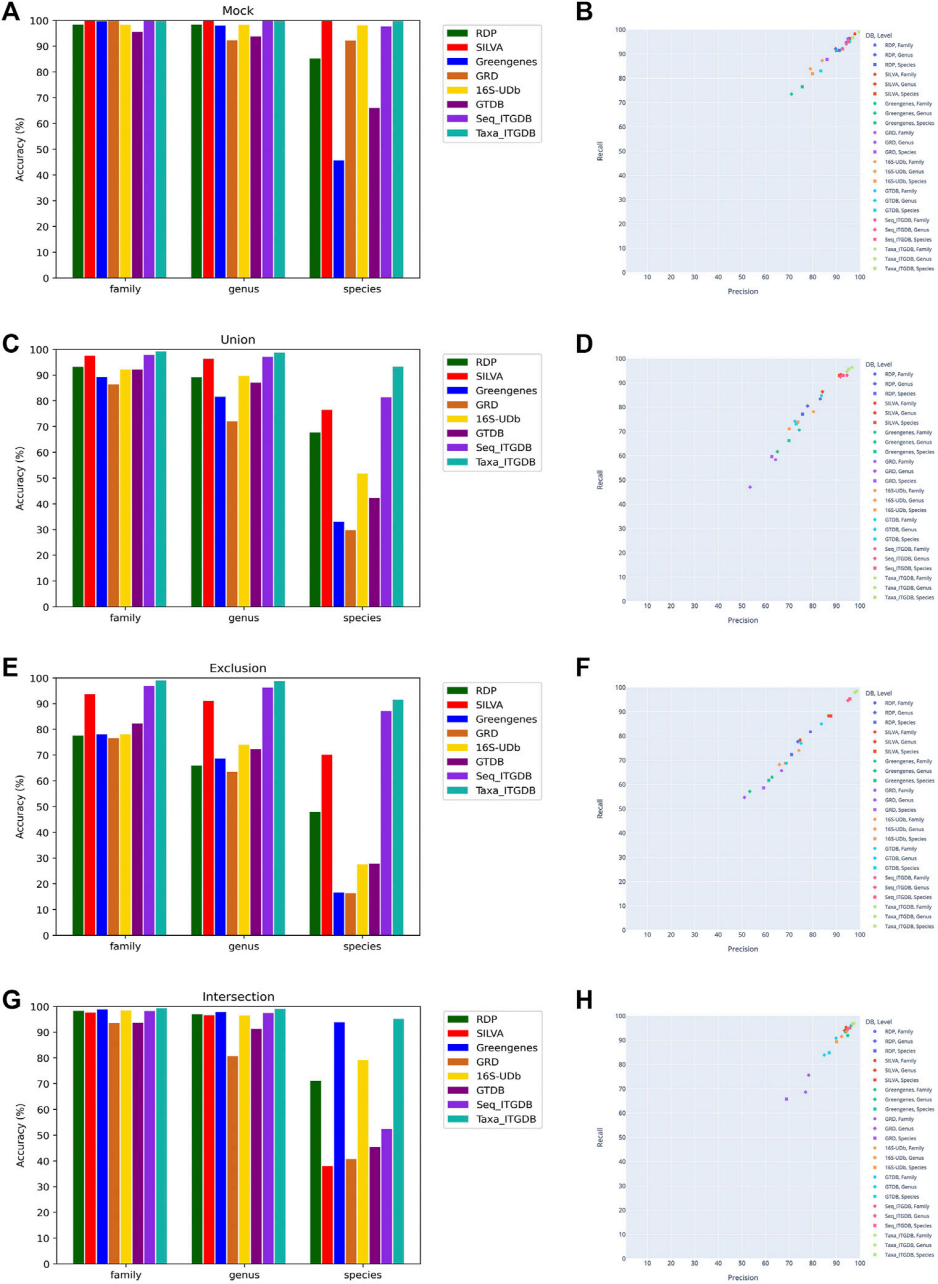
Performance comparison of using different reference databases to classify sequences in the combined mock community, Union, Exclusion, and Intersection datasets. Accuracy of classifying the mock community **(A)**, Union dataset **(C)**, Exclusion dataset **(E)**, and Intersection dataset **(G)**. Precision and recall of classifying the mock community **(B)**, Union dataset **(D)**, Exclusion dataset **(F)**, and Intersection dataset **(H)**.

The accuracy results of all databases using the mock community, Union, Exclusion, and Intersection validation datasets are shown in Figure 3A, Figure 3C, Figure 3E, and Figure 3G. In Figure 3, the taxonomy-based ITGDB had the highest accuracy at the family, genus, and species levels in all the validation datasets, while the sequence-based ITGDB had the second highest accuracy in the Union and Exclusion test cases. When compared with RDP, SILVA, Greengenes, GRD, 16S-UDb, and GTDB, the taxonomy-based ITGDB had at least 16, 21, and 1% higher accuracy than the above databases at the species level in Union, Exclusion, and Intersection datasets, respectively.

The results of accuracy, precision, recall, and F1-score of the different databases are shown in Table 2. The scatter plots in Figure 3B, Figure 3D, Figure 3F, and Figure 3H illustrate precision and recall for each reference database. The taxonomy-based ITGDB also showed the best performance in all the validation datasets. For the mock community, SILVA’s performance was in the second place in most of the validation metrics. For Union and Exclusion datasets, sequence-based ITGDB demonstrated the second-best performance in all the validation metrics. The accuracy difference between the ITGDBs and SILVA became larger in the Exclusion dataset than Union because ITGDBs contained more complete taxonomies than SILVA. For the Intersection dataset, Greengenes and sequence-based ITGDB were in the second place in most of the validation metrics. Greengenes did not show good performance in the mock community, Union, and Exclusion datasets, but inversely demonstrated accuracy similar to the taxonomy-based ITGDB in the Intersection dataset.

**TABLE 2:** Performance comparison between different 16S rRNA databases. The bold font and underline symbol indicate the highest and the second highest value, respectively.

Dataset	Metrics	Level	RDP	SILVA	Greengenes	GRD^a^	16S-UDb	GTDB^b^	Seq_ITGDB^c^	Taxa_ITGDB^d^
Mock	Accuracy (%)	F^e^	98.30	99.88	99.65	99.81	98.24	95.51	**99.89**	99.88
		G^f^	98.29	99.85	98.01	92.27	98.23	93.77	**99.86**	99.85
		S^g^	85.18	**99.77**	45.64	92.21	98.07	66.04	97.63	**99.77**
	Precision (%)	F	95.58	97.83	89.96	94.88	84.01	95.76	94.22	**99.69**
		G	89.71	95.65	71.00	94.24	79.03	92.55	95.08	**99.98**
		S	91.26	95.23	75.57	86.06	79.89	83.41	92.64	**96.95**
	Recall (%)	F	96.53	98.25	91.29	96.23	87.33	95.19	94.04	**99.13**
		G	92.15	96.05	73.50	94.81	83.92	92.17	95.27	**99.01**
		S	91.45	94.96	76.51	87.76	81.92	83.06	92.04	**96.62**
	F1-score (%)	F	96.00	98.01	90.16	95.37	85.04	94.67	93.91	**99.25**
		G	89.93	95.83	71.79	94.47	80.07	91.54	94.78	**99.33**
		S	90.27	94.98	75.09	86.59	80.54	83.06	92.08	**96.68**
Union	Accuracy (%)	F	93.12	97.46	89.24	86.40	92.20	92.11	97.81	**99.19**
		G	89.18	96.35	81.56	72.04	89.66	87.06	97.08	**98.77**
		S	67.74	76.52	33.02	29.80	51.75	42.21	81.35	**93.23**
	Precision (%)	F	83.19	91.82	74.30	64.27	80.32	83.70	94.50	**96.67**
		G	77.81	84.14	65.03	53.47	70.03	72.44	91.72	**94.55**
		S	75.68	91.32	69.93	62.68	73.71	73.00	92.87	**95.27**
	Recall (%)	F	83.40	93.5	70.59	58.36	78.15	84.74	93.14	**96.37**
		G	80.47	86.35	61.66	47.05	71.06	74.17	92.53	**94.72**
		S	77.14	93.00	66.20	59.65	73.85	73.06	93.07	**95.74**
	F1-score (%)	F	82.71	92.10	70.56	58.96	77.57	82.90	93.33	**96.27**
		G	78.21	84.60	61.40	46.82	68.71	71.49	91.34	**94.36**
		S	75.67	91.61	67.09	59.60	73.01	72.19	92.6	**95.30**
Exclusion	Accuracy (%)	F	77.57	93.63	78.07	76.60	78.04	82.24	96.87	**99.06**
		G	65.94	91.02	68.68	63.46	73.99	72.29	96.26	**98.66**
		S	47.98	70.18	16.59	16.35	27.54	27.89	87.06	**91.45**
	Precision (%)	F	79.04	86.71	62.77	66.78	74.12	83.68	95.43	**98.63**
		G	73.69	74.66	53.34	51.08	65.89	75.05	94.92	**97.94**
		S	71.03	87.63	61.38	59.20	68.44	68.80	95.77	**97.97**
	Recall (%)	F	81.74	88.31	63.03	65.72	74.03	84.97	95.11	**98.49**
		G	77.65	78.33	57.17	54.70	68.25	77.00	94.60	**98.02**
		S	72.39	88.27	61.73	58.64	68.82	68.75	95.32	**97.97**
	F1-score (%)	F	78.73	86.29	60.75	64.53	72.69	82.94	94.89	**98.41**
		G	74.20	75.43	53.52	51.25	65.87	74.88	94.28	**97.78**
		S	70.95	87.39	60.34	57.79	68.01	68.30	95.36	**97.89**
Intersection	Accuracy (%)	F	98.25	97.54	98.82	93.56	98.43	93.67	98.13	**99.33**
		G	96.93	96.57	97.82	80.67	96.52	91.25	97.43	**98.97**
		S	71.10	37.94	93.83	40.64	79.17	45.37	52.39	**95.20**
	Precision (%)	F	94.36	94.14	96.26	78.28	94.48	89.89	94.99	**97.40**
		G	93.74	93.49	93.87	76.97	92.22	84.90	94.81	**96.91**
		S	93.97	94.69	94.82	68.88	90.08	86.97	95.72	**96.87**
	Recall (%)	F	94.25	95.33	96.11	75.64	93.64	90.89	94.93	**96.99**
		G	94.07	93.98	93.45	68.63	91.57	83.93	94.75	**96.78**
		S	93.56	94.80	91.96	65.80	89.42	84.87	95.04	**96.72**
	F1-score (%)	F	94.01	94.29	96.07	75.97	93.49	89.42	94.46	**96.98**
		G	93.67	93.08	93.44	69.63	91.12	83.00	94.29	**96.53**
		S	93.32	94.06	92.88	65.14	89.11	84.96	94.96	**96.38**

a GRD, genomic-based 16S rRNA database.

b GTDB, genome taxonomy database.

c Seq_ITGDB, sequence-based integrated database.

d Taxa_ITGDB, taxonomy-based integrated database.

e F, family.

f G, genus.

g S, species.

As in Table 2 and Figure 3, 16S-UDb and GRD showed good performance on mock community classification. GRD had higher accuracy, precision, recall, and F1-score than 16S-UDb. However, for Union, Exclusion, and Intersection datasets, the trend was shown inversely that 16S-UDb had better performance than GRD. GRD did not demonstrate good accuracy at the family, genus, and species levels in Union and Exclusion datasets. GTDB did not have good accuracy at the species level in all the test cases.

**FIGURE 4:**
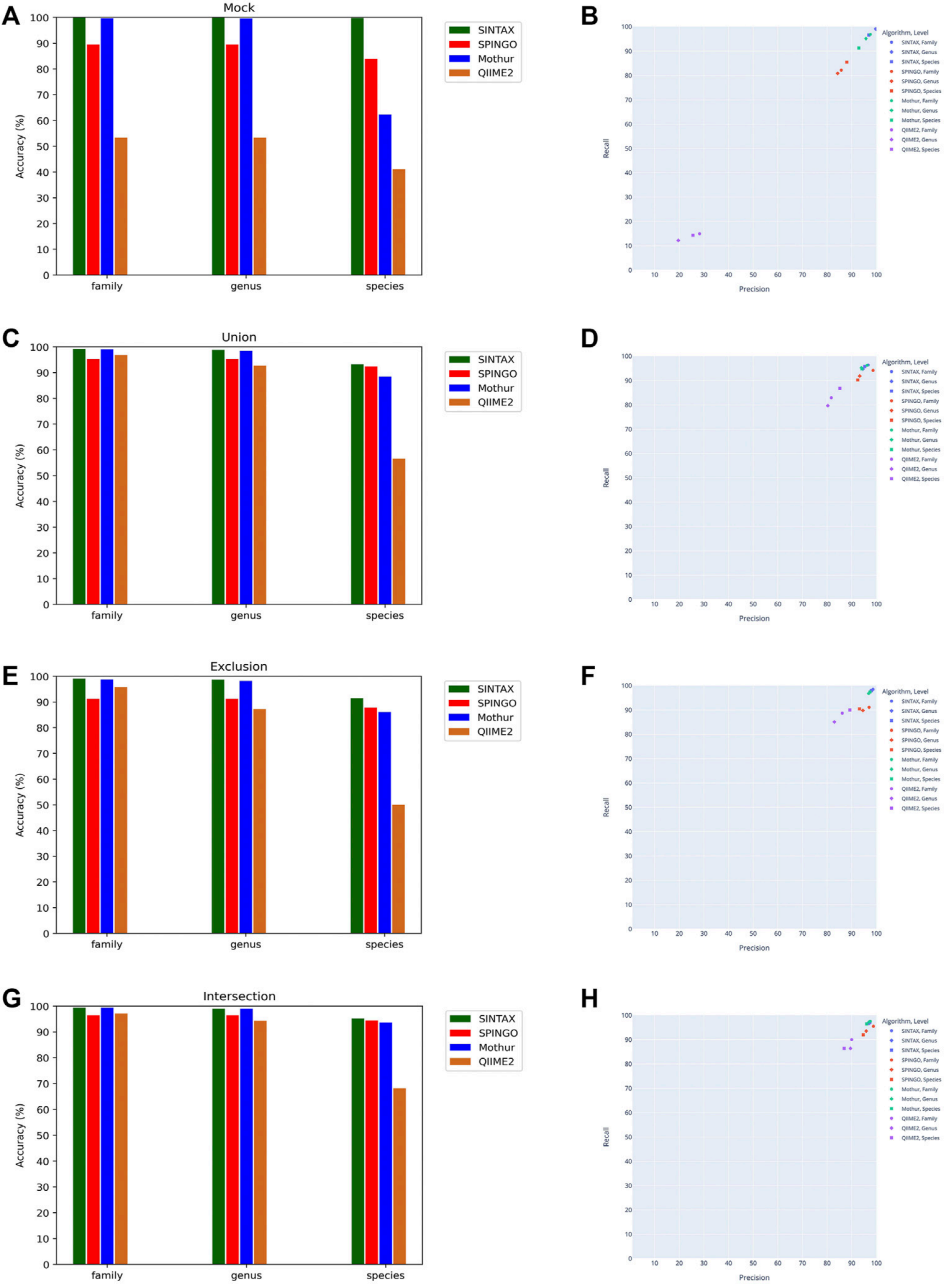
Performance comparison of different classifiers using the taxonomy-based ITGDB as the reference database to classify the sequences in mock community, Union, Exclusion, and Intersection datasets. Accuracy of classifying the mock community **(A)**, Union dataset **(C)**, Exclusion dataset **(E)**, and Intersection dataset **(G)**. Precision and recall of classifying the mock community **(B)**, Union dataset **(D)**, Exclusion dataset **(F)**, and Intersection dataset **(H)**.

Since the taxonomy-based ITGDB showed the best performance in the database comparison analysis, we further used the taxonomy-based ITGDB to compare the accuracy with different 16S rRNA classifiers, as shown in Figure 4 and Table 3. SINTAX and Mothur showed similar accuracy at the family and genus levels (Figure 4). For species level assignment, SINTAX and SPINGO had an accuracy of more than 80% in all the validation datasets. QIIME2 had lower accuracy in all the validation datasets. For the mock community dataset, SINTAX demonstrated the best performance in most of the validation metrics (Figures 4A,B; Table 3). For the Union dataset, SINTAX showed the best performance at species level assignment, while Mothur was in the second place in most of the metrics (Figure 4C, Figure 4D, and Table 3). For the Exclusion dataset, SINTAX had the highest scores in all the validation metrics. The Mothur classifier was in the second place in most of the metrics in the Exclusion dataset (Figure 4E, Figure 4F, and Table 3). For the Intersection dataset, SINTAX, SPINGO, and Mothur had accuracy more than 90%. Both SINTAX and Mothur possessed the best or the second best in most of the metrics (Figure 4G, Figure 4H, and Table 3).

**TABLE 3:** Performance comparison between different classifiers using the taxonomy-based integrated database. The bold font and underline symbol indicate the highest and the second highest value, respectively.

Dataset	Metrics	Level	SINTAX	SPINGO	Mothur	QIIME2
Mock	Accuracy (%)	F^a^	**99.88**	89.50	99.71	46.64
		G^b^	**99.85**	89.48	99.63	46.61
		S^c^	**99.77**	83.93	62.36	39.22
	Precision (%)	F	**99.69**	85.76	97.57	98.77
		G	**99.98**	84.29	95.78	98.05
		S	96.95	87.99	92.88	**97.20**
	Recall (%)	F	**99.13**	82.19	96.89	86.78
		G	**99.01**	80.86	95.15	89.68
		S	**96.62**	85.48	91.31	85.73
	F1-score (%)	F	**99.25**	82.78	96.97	90.59
		G	**99.33**	81.40	95.20	92.41
		S	**96.68**	85.91	91.74	89.45
Union	Accuracy (%)	F	**99.19**	95.22	99.01	99.16
		G	**98.77**	95.22	98.50	98.60
		S	**93.23**	92.35	88.48	83.09
	Precision (%)	F	96.67	**98.65**	96.23	97.64
		G	94.55	93.23	93.92	**95.19**
		S	95.27	92.44	94.13	**95.64**
	Recall (%)	F	96.37	94.18	96.23	**98.11**
		G	94.72	91.85	95.21	**96.96**
		S	95.74	90.22	94.75	**96.08**
	F1-score (%)	F	96.27	96.01	95.97	**97.63**
		G	94.36	91.48	94.02	**95.59**
		S	95.30	90.76	94.20	**95.71**
Exclusion	Accuracy (%)	F	99.06	91.21	98.74	**99.22**
		G	**98.66**	91.21	98.21	98.49
		S	**91.45**	87.81	86.05	67.29
	Precision (%)	F	**98.63**	97.05	97.34	98.54
		G	97.94	94.54	96.89	**98.56**
		S	97.97	93.14	97.42	**98.32**
	Recall (%)	F	98.49	91.09	97.45	**98.87**
		G	98.02	89.81	96.81	**98.81**
		S	97.97	90.47	97.40	**98.52**
	F1-score (%)	F	98.41	93.41	97.24	**98.57**
		G	97.78	91.31	96.62	**98.51**
		S	97.89	91.38	97.31	**98.34**
Intersection	Accuracy (%)	F	**99.33**	96.41	99.28	99.27
		G	98.97	96.41	**98.97**	98.96
		S	**95.20**	94.34	93.56	91.80
	Precision (%)	F	97.40	**98.80**	97.57	97.72
		G	96.91	95.89	97.07	**97.12**
		S	**96.87**	94.68	96.06	96.62
	Recall (%)	F	96.99	95.51	97.55	**97.62**
		G	96.78	93.54	97.33	**97.42**
		S	96.72	92.01	96.48	**96.90**
	F1-score (%)	F	96.98	96.91	97.44	**97.50**
		G	96.53	94.33	96.86	**96.95**
		S	96.38	92.93	95.92	**96.70**

a F, family.

b G, genus.

c S, species.

Setting a confidence threshold for full-length sequence assignment can limit a classifier’s performance. The comparison results of using “Confidence threshold” and “No confidence threshold” settings in SINTAX are shown in Table 4. When setting the confidence threshold (default = 0.8) to limit the assignment depth, less than 50% of the sequences in Union, Exclusion, and Intersection datasets could be assigned at the species level. Conversely, when classifying the sequences without limitation, more than 99% of the sequences of all the validation datasets could be assigned to the species level, and most of the sequences were correctly assigned (Figure 3 and Table 2).

**TABLE 4:** The comparison of assignment depth using the taxonomy-based ITGDB with and without application of a confidence threshold.

Dataset	Type	Family (%)^a^	Genus (%)^a^	Species (%)^a^
Mock	No threshold	100.00	100.00	100.00
	Threshold	99.41	97.97	87.02
Union	No threshold	99.87	99.87	99.87
	Threshold	71.12	67.34	38.25
Exclusion	No threshold	99.79	99.79	99.79
	Threshold	80.25	75.39	48.86
Intersection	No threshold	99.91	99.91	99.91
	Threshold	65.63	63.94	43.48

a Numbers indicate the percentage of sequences assigned to the corresponding taxonomic levels.

## 4 Discussion

In this study, we proposed two types of 16S rRNA integrated databases for prokaryotic sequence classification—taxonomy-based integration and sequence-based integration databases. The taxonomy-based integration database, assembled by collecting the sequences with exact species names and then integrating all the unique sequences from RDP, SILVA, and Greengenes, showed the best performance in most of the validation metrics.

Reasons of the taxonomy-based integration database with the best performance are discussed below. In this study, sequence-based integration collected all the sequences from RDP, SILVA, and Greengenes without taking taxonomic annotation quality into consideration, which was used to show that only collecting all the sequences could not give promised performance. Sequence-based integration included more sequences than taxonomy-based integration. Intuitively, a database with more reference sequences might provide better classification performance. However, if the collected sequences were annotated with ambiguous taxonomy names or only had low taxonomic depth information (e.g., only included taxonomic information down to the phylum, class, or order level), the blurred sequences limit a classifier’s performance (Lan et al., 2012). This situation could be observed from Figure 3 and Table 2 when comparing the performance between taxonomy-based ITGDB and sequence-based ITGDB. Only integrating all 16S sequences could not guarantee the classification performance. Therefore, taxonomy-based integration is suggested for application.

In the past, NGS platforms sequenced part of the 16S rRNA hypervariable regions to identify the species to which a sample belonged. These sequenced regions included approximately 200 ∼500 nucleotides. The 16S rRNA classifiers set their confidence thresholds to prevent the over-classification issue based on these short reads. Previous studies reported that in order to assign a sequence to the genus level accurately, the sequence length needs to be at least 400 nucleotides (Okubo et al., 2009), and a full-length sequence could provide taxonomic resolution to the species level (Jeong et al., 2021). Notice that the 16S rRNA full-length sequences include approximately 1,500 ∼1,600 nucleotides (Nossa et al., 2010; Wagner et al., 2016). Since our classification target was the prokaryotic 16S full-length sequences, we found that using confidence thresholds to limit the taxonomic assignment depth made the prediction too conservative to reach the species level (Table 4). Therefore, the “no cut-off” assignment results were applied in our analyses.

**FIGURE 5:**
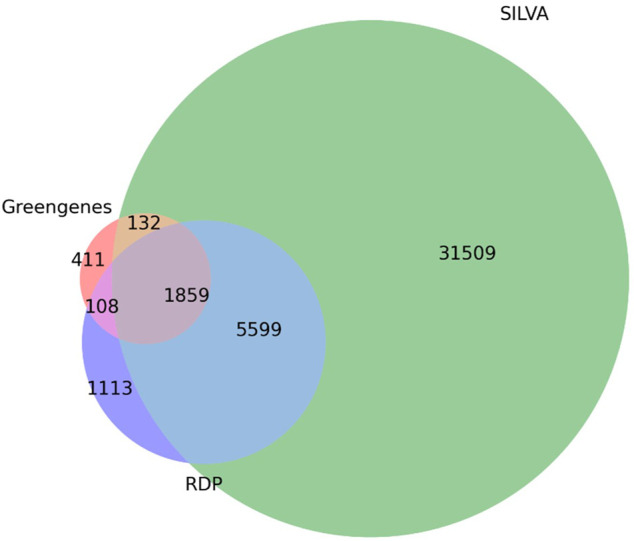
Species distribution of RDP, SILVA, and Greengenes databases. The Venn diagram depicts the number of shared and unique species between the three databases.

The database comparison analyses indicated that the taxonomy-based ITGDB had the best performance. In the Union dataset, the taxonomy-based ITGDB showed better accuracy than other databases, especially at the species level. There were two factors that explain why the taxonomy-based ITGDB could identify most of the species. One was that the taxonomy-based ITGDB covered all of the available species of RDP, SILVA, and Greengenes. The other was that the taxonomy-based ITGDB removed a considerable number of anomalous sequences by only integrating the sequences with exact species names. The Venn diagram in Figure 5 investigates the unique species names collected in RDP, SILVA, and Greengenes. The unique species taxonomies in RDP, SILVA, and Greengenes were 1,113, 31,509, and 411, respectively. Greengenes included the smallest number of species labels because this database had not been updated for many years, which was also the reason why Greengenes had the lowest performance among all the databases. However, Greengenes showed good performance with the Intersection dataset (the second highest scores in most of the metrics) because this dataset did not have unique taxonomy (the taxonomies only exist in one of RDP, SILVA, and Greengenes).

The sequence-based ITGDB ranked second in accuracy when using the Union and Exclusion datasets for validation (Table 2). However, the accuracy performance of the sequence-based ITGDB became worse than RDP and Greengenes with the Intersection dataset. This situation indicated that simply collecting more sequences could not enhance the classification performance. The reason why the sequence-based ITGDB performed well with the Union and Exclusion datasets was that the sequence-based ITGDB included all the available taxonomies from RDP, SILVA, and Greengenes to overcome the unique taxonomy issue. However, collecting all the available sequences also meant having more sequences with low resolution taxonomies. Namely, the information at the species level did not have an exact species name, which could interfere with the taxonomic assignment procedure (Xue et al., 2022). This shortcoming was exposed when the validation dataset did not have unique taxonomy issues (e.g., the Intersection dataset).

The sequence-based ITGDB showed better performance than SILVA with the Intersection dataset because the sequence-based ITGDB collected longer sequences under the same taxonomies. This might be the reason why the sequence-based ITGDB could identify the sequences more accurately than the SILVA database (Karagöz and Nalbantoglu, 2021). The reason why SILVA had better performance than Greengenes and RDP with the Union and Exclusion datasets, but lower performance with the Intersection dataset, was similar to the reasons outlined above for the sequence-based ITGDB.

RDP had the smallest number of sequences, but it contained better curated sequences and taxonomies than SILVA (Edgar R., 2018), with 94.86% of sequences in RDP having taxonomic resolution at the species level. This could be the reason why RDP showed better performance than SILVA with the Intersection dataset. However, RDP included much less unique taxonomy than SILVA, and this prevented RDP from having better performance than SILVA with the Union and Exclusion datasets. For mock community validation, the reason why SILVA had better performance than RDP might be that SILVA included much more sequences than RDP. More reference reads allow SILVA to identify the type strain sequences more efficiently.

Greengenes did not perform well in most of the analyses. For the mock community, Union, and Exclusion datasets, Greengenes showed low accuracy at the species level because most of Greengene’s sequences did not have taxonomic resolution to the species level, and the fact that its content had not been updated for many years. It is impossible for a classifier to identify the newly discovered bacteria using Greengenes as a reference database.

The 16S-UDb had mediocre performance among the test cases. Two reasons may explain that 16S-UDb had lower performance than taxonomy-based ITGDB, especially for the species level assignment. One was that 16S-UDb collected the 97% OTU clustering sequences from RDP, SILVA, and Greengenes, which may put the sequences of different species into the same cluster and lost considerable taxonomies and reference sequences (Edgar RC., 2018; Chiarello et al., 2022). Inversely, taxonomy-based ITGDB applied 99% OTU clustering sequences from the reference databases to retain the taxonomies and sequences, ensuring taxonomy-based ITGDB could have better classification ability. Another reason was that 16S-UDb was built based on the older version of SILVA, Greengenes, and RDP, which meant it lacked the newly updated taxonomies. In Figure 3 and Table 2, 16S-UDb had better performance with the mock community and Intersection datasets than with the Union and Exclusion datasets because the mock community and Intersection datasets did not include unique taxonomies. Each sequence in 16S-UDb was full-length and with an exact species name, which could provide good performance of identifying the type-strain sequences in mock community and non-unique taxonomies in the Intersection dataset. Inversely, the Exclusion and Union datasets included a large number of unique taxonomies, which exposed the shortcoming that 16S-UDb did not collect enough reference sequences and taxonomies.

GRD also identified the sequences of the mock communities quite well, but had worse performance than 16S-UDb, when classifying the sequences of the Intersection dataset. The collected species number of GRD and 16S-UDb was 2,603 and 7,399, respectively. The difference of the collected species number might be the reason why 16S-UDb could have better ability to overcome the unique taxonomy issues than GRD when classifying the sequences of the Union, Exclusion, and Intersection datasets.

GTDB did not have good performance at the species level. Reasons for this phenomenon were that many sequences in the GTDB dataset did not have exact species names (only showed “sp [number]” at the species level) because some metagenomics assembled genomes did not include 16S gene fragments (Alishum, 2021), which interfered the performance of the classification algorithm.

By observing the number of full-length sequences (V1-V9) in Table 1, the database performance comparison in Table 2, and the species Venn diagram in Figure 5, we found that taxonomy-based ITGDB did not possess the largest number of full-length sequences (Table 1) but had the best performance in all the validation datasets (Table 2). Inversely, sequence-based ITGDB and SILVA had the largest and the second largest number of full-length sequences (Table 1) but did not have the highest scores in all the test cases. This situation indicates that large quantity of full-length sequences alone could not ensure good assignment results. The completeness of taxonomy information also needs to be considered. A large proportion of sequences without exact species names limited the classification performance of sequence-based ITGDB and SILVA. Since taxonomy-based ITGDB included all the taxonomies of RDP, SILVA, and Greengenes and each sequence was assigned with an exact species name, this is the reason why taxonomy-based ITGDB could have the best performance in all the validation datasets. In summary, taking reference sequence count, taxonomy completeness, and taxonomy count into consideration could enhance a sequence classifier’s taxonomic resolution.

Analyses of the ITGDBs’ performance with different classifiers demonstrated that the taxonomy-based ITGDB could work well with several widely used classifiers. For the mock community dataset, SINTAX showed the best performance at the family, genus, and species levels (Figure 4). For the Union, Exclusion, and Intersection datasets, SINTAX, SPINGO, and Mothur showed good performance at all the taxonomic levels. QIIME2 had lower accuracy in all the test cases. We found that the QIIME2 classifier worked normally when classifying the sequences of HMP and Zymo mocks but did not work well with Mockrobiota sequences (97% Mockrobiota sequences were classified as “Spiroplasma mirum” species). However, other classifiers, SINTAX, SPINGO, and Mothur, did not have such a problem. Therefore, for species-level assignment, SINTAX, SPINGO, and Mothur are suggested to be used with taxonomy-based ITGDB.

## 5 Conclusion

This work proposed two types of 16S rRNA integrated databases—sequence-based integration and taxonomy-based integration. The experimental results showed that taxonomy-based integration provided better performance and could work well with the widely used 16S rRNA classifiers. The proposed databases can support full-length 16S rRNA classification and enhance the taxonomic resolution to the species level.

## Data Availability

The datasets presented in this study can be found in online repositories. The names of the repository/repositories and accession number(s) can be found in the article/Supplementary Material.
